# Lactose Intolerance: What Your Breath Can Tell You

**DOI:** 10.3390/diagnostics10060412

**Published:** 2020-06-17

**Authors:** Luelle Robles, Ronny Priefer

**Affiliations:** Department of Pharmaceutical Sciences, Massachusetts College of Pharmacy and Health Sciences University, Boston, MA 02115, USA; lrobl1@stu.mcphs.edu

**Keywords:** lactose intolerance, lactase-phlorizin hydrolase, lactose malabsorption, lactose tolerance test, hydrogen breath test

## Abstract

Lactose intolerance has seen recognized as a clinical syndrome characterized by pain, abdominal distention, flatulence, and diarrhea after the consumption of lactose. Lactose is a common disaccharide found in dairy that requires lactase-phlorizin hydrolase (LPH) to break down into glucose and galactose. A deficiency in this enzyme results in flora bacteria further along in the gastrointestinal tract (GI) tract to metabolize the excess lactose to numerous gases, including H_2_. Recent studies show that the risk of symptoms after lactose ingestion depends on the dose of lactose, LPH expression, intestinal flora, and sensitivity of the gastrointestinal tract. Currently, there are several diagnostic tests that investigate the biological mechanism of lactose intolerance such as blood, biopsy, genetic, and breath tests. Due to its relatively low cost, availability, and non-invasiveness, the hydrogen breath test (HBT) has become a popular technology to aid in the diagnosis of many gastroenterological diseases, specifically lactose intolerance. Additionally, while administering the HBT there seems to be a lack of uniform criteria amongst the various studies, with many using their own guidelines, which may in turn cause inconsistency with the analysis of the results. With ever improving nanotechnology, novel approaches to expedite and lower the costs of the HBT has become an area of research with significant advancements.

## 1. Introduction

Milk is a significant component of our diet containing a high nutritive value that is fundamental in the nourishment of humans beginning from the first days of life onward. Milk contains lactose and other crucial oligosaccharides that help in the development of probiotic bacteria in the human intestine which protects the GI tract from unwanted bacteria and the development of certain infections [1]. However, many people are unable to properly digest lactose. Lactose intolerance was first described by Hippocrates around 400 years B.C., although the clinical symptoms have only truly been recognized in the last 50 years [2,3]. The prevalence of lactose intolerance varies by community and ethnic group. In South America, Africa, and Asia, rates of lactose intolerance exceed 50% [4,5]. In the United States, the prevalence is reported to be 15% among Caucasians, 53% among Hispanic Americans, and 80% among Americans of African ancestry [4,6]. 

**Figure 1:**
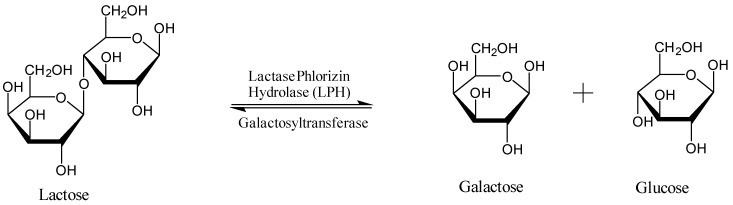
Enzymatic hydrolysis and synthesis of lactose.

Lactose, the main source of sugar from mammalian milk products, is a disaccharide consisting of d-glucose and d-galactose held together by a β-1,4 glycosidic bond (Figure 1) [1,7]. Synthesis of lactose in the mammary gland occurs via an activated uridine di-phosphate galactose being coupled with glucose by galactosyl transferase [7]. In order for lactose to be digested, lactase phlorizin hydrolase (LPH) breaks down the disaccharide into glucose and galactose, which are then carried via the sodium glucose transporters across the intestinal brush border [7]. LPH is found on the upper surface of enterocytes on the microvilli of the small intestine and is largely expressed in the central jejunum. Once lactose is hydrolyzed, the monosaccharides are individually utilized by the body; glucose as a source of energy, galactose as part of glycoproteins and glycolipids [1,7,8].

**Figure 2:**
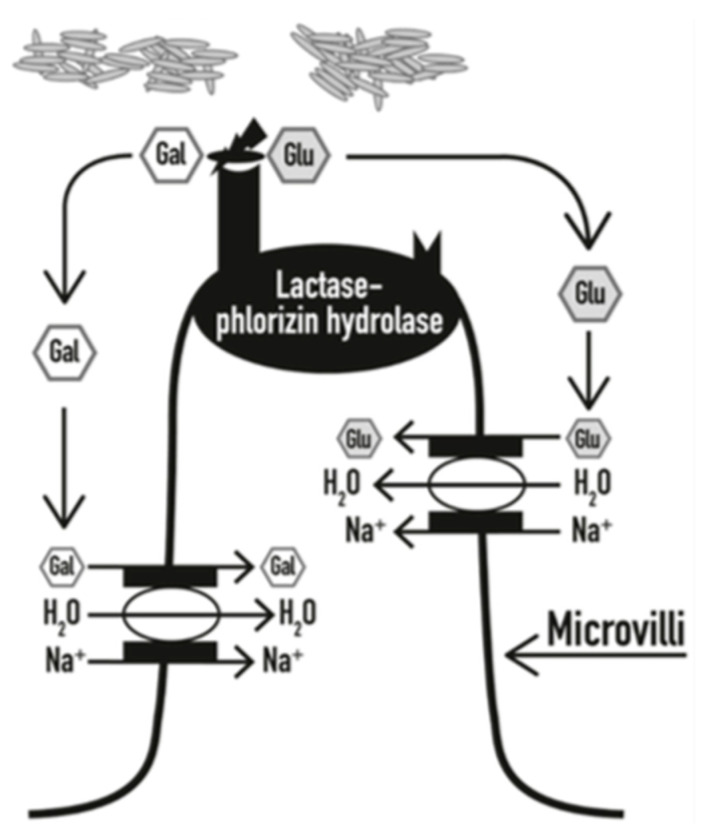
Lactose hydrolysis and rapid absorption at the brush border of the small intestine (as taken from [1]).

The development of lactose intolerance has been widely studied and recognized due to many patients presenting with symptoms related to the consumption of lactose-containing foods. Lactose intolerance is primarily due to the impaired production of LPH. Generally, lactase activity is used to measure digestive capacity, so a deficiency results in unabsorbed lactose being present in a person’s intestinal tract leading to susceptible individuals developing gastrointestinal symptoms known as hypolactasia [9]. As undigested lactose passes through the small intestine, it will set up an osmotic gradient across the gut wall causing an influx of water (Figure 2) [10,11]. The lactose then continues unchanged to the large intestine where the intestinal flora cleaves lactose into short chain fatty acids and gases (primarily hydrogen, carbon dioxide, and methane) [8,12,13]. The influx of water causes diarrhea, while the gas byproducts can lead to abdominal pain, bloating, and flatulence for several hours after digesting lactose-containing products. Additionally, symptoms of lactose intolerance may also appear systemically, presenting as headaches, inability to concentrate, fatigue, joint/muscle pain, and mouth ulcers. Symptoms of lactose intolerance typically do not occur until there is less than 50% of lactase activity. Although lactase expression is not upregulated by lactose digestion, tolerance can also be induced by adaptation of the intestinal flora [8,14].

According to the U.S. National Library of Medicine, about 65% of the human population, undergoes a decrease in lactase activity after infancy. If there is a suspected large deficiency in the lactase enzyme, one’s levels are observed in comparison to levels of an infant when lactase activity is at its highest. Whatever the causes of the deficiencies, affected individuals can be classified into primary or secondary lactase deficiency. Primary lactase deficiency (#MIM223100) is regarded as a common genetic condition resulting in a developmentally regulated change where LPH production decreases as one’s diet becomes less reliant on dairy products. Secondary lactase persistence is a condition derived from intestinal damage secondary to multiple diseases caused by gut trauma due to surgery, infections, or disease that may have caused damage to the gastrointestinal tract (GI) tract such as celiac disease or Crohn’s disease [10,15,16]. Additionally, there is an extremely rare type of lactase deficiency known as congenital lactase deficiency (CLD). CLD (#MIM223000) is a severe autosomal recessive disease caused by absent or reduced levels of lactase expression from birth deriving from a mutation in the LPH gene. As a result of this mutation, CLD exhibits an onset of watery diarrhea after the start of breastfeeding [11,16,17,18]. All types of lactase deficiencies can be the root cause of lactose intolerance symptoms due to an insufficient level of lactase activity in the brush border of the small intestine [19].

Lactase deficiency and lactose malabsorption is strongly proportional to ethnicity. Caucasians of Northern Europe descent are known to have lactase persistence, retaining a high level of activity of LPH that is present through all of adulthood. Lactase persistence is useful in society as it allows for the incorporation of milk from domesticated animals into our diets. Conversely, the majority of the rest of the world such as the Asian, Native American, and African American populations, as well as Caucasian populations with a polymorphism of a 13910C/T variant, have lactase non-persistence (hypolactasia). Though the single nucleotide polymorphisms (SNP) variant of 13910C/T is more known and studied, there are a variety of other SNPs associated with different ethnic groups, such as 13915T > G, 14010G > C, 22018G > A, and 13907 C > G [8]. Lactase non-persistence is an ancestral trait characterized as a wild-type condition that most individuals have reduced lactase activity at the jejunal border after breastfeeding [1]. It is reported that the highest rates of lactose malabsorption exist in the Asian, Native American, and African American populations, while the lowest rates are reported in people of Northern European origin and the white population of the United States [20]. Based on lactase deficiency and lactose malabsorption of the various ethnic groups, it is determined that deficiency and malabsorption also correlate with one another.

With all the diagnostic techniques available for the testing of lactose intolerance, there seems to be a lack of uniform criteria that each procedure follows. Variations in criteria, especially for the hydrogen breath test (HBT), can affect results that can potentially lead to different interpretations of lactose malabsorption and symptoms of lactose intolerance.

## 2. Types of Diagnostic Testing for Lactose Intolerance

Currently, there are four methods that are available in the diagnosis of lactose malabsorption and lactose intolerance. Many people self-report their lactose intolerance, which refers to a person’s belief that they suffer from gastrointestinal symptoms after consuming lactose. Since self-reported lactose intolerance does not include the involvement of clinical tests, it is clear that self-reported lactose intolerance is an unreliable diagnostic tool for lactose malabsorption [7,20]. Therefore, the use of the four diagnostic methods that will be mentioned are used for the proper analysis of lactose intolerance.

The first type is to test the lactase activity at the jejunum by obtaining biopic fragments of the small intestine mucous membrane and measuring the activity of LPH. This test is considered the reference standard for primary lactase deficiency and has an advantage that both endoscopy and biopsy are utilized. This method can be used to exclude other conditions that cause secondary lactase deficiency which damage the GI tract. Biopsy of the jejunum is rarely used due to invasiveness and high cost, as it is the most expensive of all the diagnostic tools. The biopsy has the possibility of being influenced by the unexpected delivery of lactase to the small intestine [1,8]. This test rarely causes false positives, but false negatives may occur due to a patchy enzyme expression.

Secondly, a genetic test can be used to establish lactase non-persistence or primary lactase deficiency in Caucasian patients with a 13910 C/T polymorphism. Due to its exclusivity to Caucasian patients, this test is not suitable for patients in other populations who may also be at increased risk of lactase deficiency. The main reason why this test is not suitable for patients of other ethnicities is due to other SNPs being present in other ethnic populations [8]. This limitation can be potentially disregarded if other genetic polymorphisms can be detected with this test. However, genetic testing should be performed whenever congenital lactase deficiency is suspected in infants with symptoms and a positive response to dietary eliminations of lactose [16,21]. Even though this test can rarely cause false positives, false negatives can occur due to causes of secondary lactose malabsorption. Therefore, conditions related to secondary lactose malabsorption should be ruled out to avoid possible false negatives. Since lactose malabsorption is considered a recessive condition, a heterozygous genotype has to be considered a negative test result.

The third test that is more common than the latter two and requires blood samples for testing is the lactose tolerance test. This test involves a lactose challenge where a standard dose of lactose is given prior to testing. The purpose is to observe a rise in blood glucose levels due to the breakdown of lactose. Blood samples are taken at various times during the test to determine plasma glucose concentrations after the administration of an oral lactose dose. Lactose intolerance is determined by a maximal plasma glucose increase of 1.1 mmol/L or less. However, results from this test may be exaggerated in patients who have variations in post prandial blood sugar levels due to diabetes or another pre-existing condition [12]. Common factors that cause false positives are rapid GI transit and impaired glucose tolerance, while false negatives can be caused by fluctuations in blood glucose levels. Due to its low sensitivity and specificity, it is rarely used today. An important limitation to be aware of is that alteration of the bowel anatomy can affect the lactose intolerance test. Though, in some instances, the lactose tolerance test may be combined with the fourth method, the hydrogen breath test (HBT). While the first three tests discussed have certain limitations and are dependent on various populations based on exclusivity, the HBT is the most commonly used due to its ease of administration, low cost, and non-invasiveness (Table 1).

## 3. The Hydrogen Breath Test (HBT)

The HBT is widely used to explore the pathophysiology of functional gastrointestinal disorders, such as lactose intolerance. HBT is the most common type of lactose intolerance test due to its low cost, non-invasiveness, and reasonably high sensitivity and specificity [22]. The use of the HBT for lactose intolerance began in the 1970s, when Newcomer and his associates studied lactose malabsorption by analyzing breath H_2_ and CO_2_ levels. Subsequently, Bond and Levitt concluded that some disaccharides remain unbroken and unabsorbed due to incomplete digestion in the small intestine. This was done through analysis of H_2_ in the breath after changes were observed in H_2_ concentrations in expired air after ingested sugar reached the colon intact and undigested [23,24,25,26]. Thus, an increase in breath hydrogen indicates maldigestion and reflects colonic fermentation of lactose. It is important to note that anatomical bowel changes that a patient may have poses a limitation for the HBT [12]. The excretion of the gases may be altered which may lead to misinterpretation of results. Of the lactose intolerance diagnostic tests, several studies have suggested that the hydrogen breath test is superior due to its ease of use and its effectiveness in understanding the atypical pathophysiology of lactose malabsorption [4].

**Figure 3:**
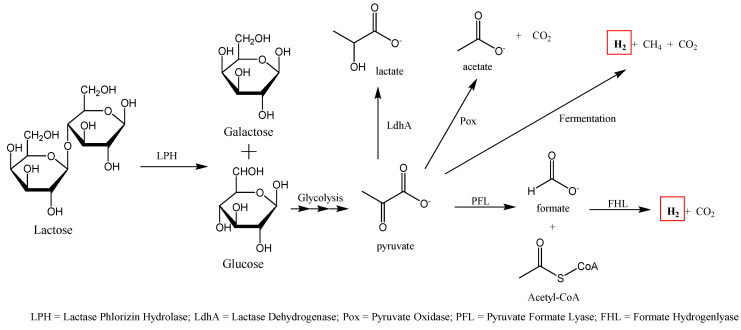
Colonic metabolism of lactose by *Escherichia coli*. Gases such as hydrogen, methane, and carbon dioxide are formed as products (modified from [27,30]). Red frame delineates H_2_ production.

The HBT is used to determine the amount of hydrogen produced through fermentation of undigested carbohydrates by colonized anaerobic bacteria located in the lower GI tract. In people who are lactase deficient, the flora in the colon produces hydrogen that can navigate into the intestinal mucosa and be absorbed into the person’s circulation and then excreted by the lungs [27]. Additionally, methane or carbon dioxide, can also be measured separately as correlation exist for severity of lactose intolerance and these gases, particularly CH_4_ (Figure 3) [28,29]. If lactose intolerance symptoms were observed and a positive breath test was determined, patients will be summarized into intensities of none, mild, or intense.

Rezaie et al. described specific guidelines and procedures that must be followed as well as certain medications or lifestyle choices prior to the test [31]. Prescription and OTC medications such as antibiotics, probiotics, promotility drugs, and laxatives should be avoided in advance of the procedure. Antibiotics should be avoided in the previous 4 weeks as they have a negative effect on the hydrogen and methane production that will be calculated in a patient’s exhaled breath because antibiotics will kill the bacteria that plays a role in the fermentation of lactose into gases. Similarly, motility drugs and laxatives should be discontinued 4 weeks in advance of the procedure due to it affecting transit time and delivery of lactose to the intestines and colon. Motility drugs and laxatives can greatly impact the levels of the gases that will be detected, which could possibly lead to false positives. False positives can also occur due to the presence of bacterial overgrowth of the small intestine [1,30]. Patients that report with false positive breath tests usually complain of symptoms directly after ingestion of a lactose oral load [8]. Additionally, non-producers are patients that have bacteria in their bowel, but the microorganism themselves have the inability to form hydrogen [12,30] that could lead to a false negative result. The setback of hydrogen non-production can be alleviated to a certain extent be examining patient reports of symptoms after the test dose is given [8]. Although, probiotics have been demonstrated to affect hydrogen levels on breath tests, there is inefficient data indicating the exact effect of halting probiotics prior to breath testing [31].

Certain lifestyle modifications must also be taken into account prior to the administration of HBT. Smoking has a significant impact, as it affects the hydrogen and carbon dioxide content of the breath, as well as causing an increase in gastric time [31,32]. Fassio et al. stated that physical activity can also have an impact on the HBT as excessive breathing during exercise affects hydrogen levels [1]. Since hyperventilation inversely affects hydrogen levels, excessive exercise should be avoided [33]. These lifestyle changes only need to be stopped on the day of and at least four hours before the HBT [1]. Additionally, patients who have small intestinal bacterial overgrowth (SIBO) will result in early fermentation and elevation of gases during a breath test performed to test lactose malabsorption. Due to this, SIBO should be ruled out to avoid any possible false positive results. If a patient does present with SIBO, then the patient cannot be included in the study due to lactose being exposed to bacteria in the small intestine that will cause inaccurate results [31]. Though, if a patient is SIBO negative or SIBO has been eradicated in patients with refractory symptoms, the HBT may be used. 

Patients who utilize the HBT also need to adhere to a certain diet, undergo fasting, and practice a few hygienic techniques. In a randomized patient-blinded study, patients needed to be on a specific low carbohydrate diet one day before and fasted the previous night [34]. A low fasting level of breath H_2_ is essential for interpreting breath test results because they are directly affected by consumption of fermentable and complex carbohydrates [31,35]. Patients were also required to use a specific mouthwash, Chlorhexidine, and brush their teeth to eliminate the chance of an early hydrogen peak due to bacteria in the mouth. Before testing, baseline hydrogen and methane levels were measured in order to compare results from the ingestion of lactose. A standard oral dose of 35–50 g of lactose was consumed and after 30 min to an hour, samples of hydrogen and methane were collected to measure if intestinal bacteria had produced elevated levels of H_2_. The initial trapping of the breath was accomplished with a Quintron Breathtracker [34]. The samples were later analyzed via gas chromatography. Though the rise in hydrogen varies, a test was deemed positive for malabsorption when a peak of hydrogen surpassed 20 ppm (parts per million). A baseline fasting breath methane level is >3–5 ppm, so if methane was greater than 10 ppm in two or more samples, a positive diagnosis was confirmed [34,36]. Intolerance was defined as an increase of >2 over baseline using a symptom score index based on the sum of intensities (0 = none, 1 = mild, 2 = intense). In Wilder–Smith’s study, a rise in hydrogen and methane levels is the most common method to show malabsorption, but does not state the normal range of methane production for the consumption of lactose [34]. However, a few other studies define a normal range of methane production >3–5 ppm and view methane as a marker of constipation or for the slowing down of gastrointestinal transit [29,36]. Hence, there is no concrete evidence that a rise in methane is relevant in the diagnosis of lactose intolerance using the HBT. 

Another study used the Quintron Microlyzer Gas Chromatograph and a lower load dose of 20–25 g compared to the usual range of 30–50 g [37]. They administered the HBT after a 24-h low fiber diet and a 12-h fasting period to determine if the patients had increased amounts of H_2_ compared to baseline. Similar to the previous study, a peak exceeding 20 ppm above baseline for two or more samples was deemed a positive indicator of lactose malabsorption. Of the 254 patients that tested positive, 142 reported symptoms during the test. In this study, having a positive result via HBT and abdominal symptoms implied a diagnosis of lactose intolerance, but being asymptomatic did not mean they were not intolerant. Patients that reported symptoms even with a negative test result were included in the study, though, the interpretation of these results could potentially mean the results were false-negatives. However, results of a negative HBT or experiencing no symptoms may suggest different effects of lactose malabsorption in the long-run or may be due to this study’s use of a lower oral load compared to what other studies have used [37]. 

However, another study utilized gas chromatography-mass spectrometry (GCMS) with a gastrolyzer and a loading dose of 50 g/250 mL of lactose solution [38]. Patients in this study fasted overnight and advised to avoid slowly absorbed carbohydrates and fiber the night before the test. Samples were taken at baseline and multiple times after ingestion of the lactose solution. The cut-off increase of hydrogen of 20 ppm above baseline was considered to be positive. Out of 2751 hydrogen tests, 839 patients were diagnosed as intolerant. However, there were 93 patients that had an initial H_2_ value greater than 20 ppm diagnosed with lactose malabsorption. Due to samples being taken at times ranging from 0–180 min after ingestion, patients with samples after long periods of time could have been disregarded from being diagnosed with malabsorption. Therefore, the results in this study highlight the relevance of the samples taken for each patient at different time intervals, illustrating that various patients can tolerate their lactose intolerance until a certain time or that they may not be considered to have lactose intolerance depending on the circumstance and researchers conducting the study [38].

In pediatric patients, most guidelines and procedures are similar to those previously mentioned, however, there are some differences in performing the HBT to determine lactose malabsorption in pediatric patients. An oral load lactose for pediatric patients is weight-based, 2 g/kg, with a maximum of 50 g [39]. Excretion of hydrogen and methane was determined every 30 min for a total of 3 h following the oral lactose load. Every 30 min, patients were asked to mention if they were feeling any lactose intolerance symptoms. Similar to the studies mentioned above, the HBT was deemed positive when a hydrogen peak concentration exceeded 20 ppm in 2 or more samples. Abdominal pain assessments and visual scales are utilized to help children communicate what they are feeling and express the intensity of pain and symptoms. It is understandable that pediatric patients do not have a fully developed organs and digestive tracts, which indicates some changes in testing procedures for this age group.

The aforementioned studies were all done based on a technology coupled with gas chromatography, however other investigations are under way with other novel approaches measuring H_2_ concentrations. In one study, they measured breath H_2_ concentrations using a device equipped with electrochemically working hydrogen cell [25]. This is a type of fuel cell device that converts chemical potential energy into electrical energy. The breath analyzer detected H_2_ concentrations up to 250 ppm after subjects ingested 50 g of oral lactose, with an aim to evaluate the sensitivity and specificity of symptoms after the ingestion. In this study, the lactose HBT was considered positive if the patient exhaled air exceeding 20 ppm above baseline at least twice and when a symptoms had a double increase in severity. Another study obtained samples using a Y-piece/direct method before and after a lactose challenge at 20-min intervals over a 240-min period. Concentrations were measured in ppm using a sensitive electrochemical cell consisting of three electrodes and a liquid electrolyte [40]. However, in this study there was a combination of a glucose response with a normal HBT that researchers believed was due to the colonic flora unable to produce H_2_.

**Figure 4:**
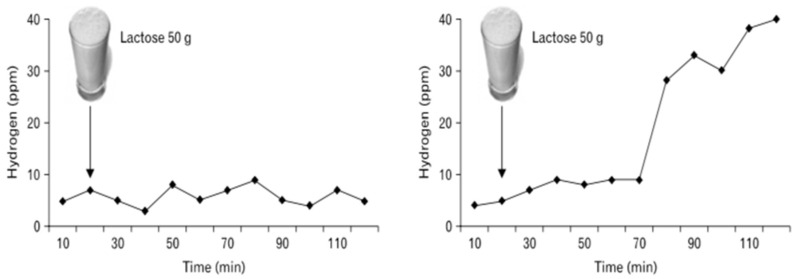
Example results from lactose malabsorption and intolerance—hydrogen breath test (HBT) with a 50 g oral lactose load. Left depicts a negative result; right depicts a positive result (as taken from [29]).

While HBT is often used, possibilities of combining the breath test with any of the other diagnostic analyses, such as the genetic test or biopsy, is a feasible idea. Though, combining the breath test with the lactose tolerance test is more common because it can test both blood spikes in glucose and increased breath hydrogen levels, both require a lactose oral dose for determination of the two. Additionally, since the HBT considers a standard lactose oral challenge dose to be given, a possibility of giving different doses ranging from 35–50 g may help determine whether a patient can tolerate a certain amount of lactose (Figure 4). This is due to the fact that the severity of lactose-induced symptoms in a patient is a function of the dose of lactose ingested and malabsorbed [14]. This could be helpful with regulating the extent of the patient’s intolerance and management options. However, all these issues depend on how an individual defines lactose intolerance and how they wish to get their symptoms to a manageable level, which is crucial in understanding the health implications of lactose intolerance.

## 4. Treatment and Management of Lactose Intolerance

Products that contain dairy are often presumed to be the cause of lactose intolerance symptoms, though there are various ways lactose intolerance can be prevented and treated. Primary treatment of lactose intolerance should be focused on controlling and improving digestive symptoms through restriction of lactose or drug therapy. Reducing the intake of any lactose-containing products is deemed as a better approach than completely removing dairy from the diet because evidence suggests that people can usually ingest up to 12–15 g of lactose in a single dose with exhibiting minimal to no symptoms [16,41]. In addition, ample removal of dairy could lead to deficiencies in essential vitamins and nutrients. A deficiency in these essential nutrients can affect bone formation or lead to bone malformations. Dairy products supply calcium and vitamin D which are essential in bone growth and maintenance in children and adults. Thus, additional supplementation is needed to supply these nutrients if no dairy consumption occurs. Therefore, a strategy to improve intolerance to lactose is adapting milk-containing foods and fermented dairy products in a patient’s diet in order for the colon to adjust. Additionally, there is a growing development of lactose-free dairy products that can still give the essential nutrients and vitamins that normal dairy products have without causing symptoms of lactose intolerance. Lactose-free dairy products use the enzyme β-galactosidase that is derived from dairy yeast for its production and goes through either a bath or aseptic process using soluble lactase enzyme. Lactose-free dairy is beneficial for lactose intolerant individuals as it allows them to enjoy dairy without experiencing the GI symptoms while also having similar nutritional effects on the human body compared to normal dairy products. Additionally, there are a variety of non-dairy products that are derived from plants and are used as alternatives such as soy, almond, coconut, and oat milk [7,8]. These non-dairy substitutes may also be fortified with calcium, vitamins D, A, B12, and riboflavin [37] and can give an equal amount of nutrients as regular milk or lactose-free milk. In a statement by the National Medical Association, lactose-free dairy products are the most ideal substitute for regular dairy products among individuals with lactose intolerance [42].

Two other options regarding the management of lactose intolerance is lactase enzyme replacement and probiotics. Lactase enzyme replacement therapy, such as Lactaid, is taken before the consumption of dairy that uses exogenous lactase to break down lactose into glucose and galactose to allow for the digestion of lactose [8,12]. Although, the use of lactase enzyme replacement is a valid therapeutic option in people with lactose intolerance, there are a variety of preparations sold over the counter and may not be equally effective to a person with intolerance. Lastly, probiotics studies have been showing evidence that they have the ability to relieve some on the symptoms of lactose intolerance due to alteration of the intestinal flora. Probiotics will stick to the intestinal lining and digest the dietary lactose that can help in the mitigation of malabsorptive symptoms [41]. Lactobacillus and bifidobacterium are types of bacteria seen in probiotics that will go through a process of fermentation [7,8,14,22]. The fermentation would allow microbial lactase to break down unabsorbed lactose by hydrolysis to its corresponding monosaccharides, opposed to making H_2_O, CO_2_, and CH_4_ [22]. In a study done, four-week consumption of probiotics with the mentioned bacteria combination improved symptoms and decreased hydrogen production in lactose intolerant patients [43]. In addition, these effects appeared to persist for at least three months after the deferment of probiotic consumption [8,44].

## 5. Conclusions

Diagnosis of lactose intolerance requires concomitant assessment of lactose digestion and abdominal symptoms, with the usage of diagnostic procedures to aid in proper identification of intolerance. Though, out of all the lactose intolerance diagnostic tests, several studies have suggested that the HBT is superior, especially to the lactose tolerance test which measures an increase in blood sugar after lactose is given as a challenge [4]. The HBT is an inexpensive, simple, and safe test that is useful in detecting the amount of hydrogen and other gases through fermentation by colonized bacteria. Based on the several studies, the HBT shows good sensitivity and excellent specificity, ranging from 76–94% and 77–96% [40]. The use of the HBT and other mediums, such as surveys or questionnaires, can further facilitate the interpretation of results. The use of other mediums with the HBT can aid in the proper diagnosis of lactose intolerance in patients, improve the interpretation of positive results associated with symptoms, and enhance the relevance and accuracy of any possible recommendations made to patients. In the various studies mentioned, the diagnosis of a positive breath test was either based on a rise in >20ppm from baseline for hydrogen or >10ppm for methane is considered a positive breath test. Though, due to many clinical studies utilizing various procedure guidelines and having different criteria for their diagnosis, it is suggested that there should be a uniform criteria that should be developed and used for the procedure and for the diagnosis of lactose intolerance. A certain oral dose, pre-procedure guidelines, and definition of a positive result with symptoms should be made definite and should not vary in clinical studies. Additionally, there seems to be more studies showing interest in using different techniques, other than gas chromatography, to interpret hydrogen concentrations. The use of an electrochemical hydrogen cell is being considered to measure breath concentrations for the diagnosis of lactose intolerance. With new ways of administering and measuring HBT, this test may remain the gold standard and may possibly put the other diagnostic tests out of favor due to ease of administration, use, and obtaining results.

## Figures and Tables

**Table 1:** Differences and similarities between the tests that are currently used for detecting lactose malabsorption/lactose intolerance (modified from [8,12]).

	Lactase Activity of Jejunum	Genetic Test	Lactose Tolerance Test	H_2_-Breath Test
**Test Principle**	Biopsy sample of the enzymatic activity of lactase enzyme	Test for the genetic 13910 C/T polymorphism	Increase in blood sugar after lactose challenge	Increase H_2_ in the respiratory air after lactose challenge
**Cut Off**	<17–20 IU/g	C:C 13910 lactase non-persistent phenotype	<1.1 mmol/L within 3 h	>20 ppm within 3 h
**Availability**	Rare	Variable	Excellent	Good
**False Positives**	Probably rare	Rare (<5%) in Caucasians	Rapid GI-transit, impaired glucose tolerance	Rapid GI-transit, small Intestinal bacterial overgrowth
**False Negative**	Patchy enzyme expression	All causes of secondary lactose malabsorption	Fluctuations in blood glucose levels	Non-H_2_-producers, full colonic adaptation
**Secondary Causes**	Can be excluded (histopathology during same procedure)	Cannot be excluded	Cannot be excluded	Cannot be excluded, kinetics of H_2_-increase can be suggestive
**Symptom Assessment**	Not possible	Not possible	Possible	Possible
**Cost**	Highest	High	Lowest	Low
**Additional Information**	Invasive and expensive	Definitive in Caucasians for primary lactase deficiency	Rarely used due to low sensitivity and specificity	Testing of choice for the diagnosis of lactose malabsorption/intolerance
